# Parental attitudes and family helmet use for all-terrain vehicles and bicycles

**DOI:** 10.1186/s40621-020-00253-2

**Published:** 2020-06-12

**Authors:** Cole Wymore, Gerene Denning, Pamela Hoogerwerf, Kristel Wetjen, Charles Jennissen

**Affiliations:** 1 Department of Emergency Medicine, Roy J. and Lucille A. Carver College of Medicine, University of Iowa, Iowa City, USA; 2 Injury Prevention and Community Outreach Program, University of Iowa Stead Family Children’s Hospital, University of Iowa, Iowa City, USA; 3 Pediatric Trauma Program, University of Iowa Stead Family Children’s Hospital, University of Iowa, Iowa City, USA; 4 Department of Pediatrics, Roy J. and Lucille A. Carver College of Medicine, University of Iowa, Iowa City, USA

**Keywords:** all-terrain vehicle, bicycle, helmet, attitudes, barriers, safety behavior

## Abstract

**Background:**

Helmets prevent head trauma in both all-terrain vehicle (ATV) and bicycle crashes. This pilot study’s objective was to compare family helmet use and participant attitudes regarding helmets for ATVs versus bicycles.

**Methods:**

A convenience sampling of adults attending a 2017 university-sponsored health fair who had at least one child < 18 years living at home were surveyed. Demographics, frequency of helmet use, and information about factors influencing helmet use were collected. Descriptive (frequencies) and bivariate (Fisher’s exact test) analyses were performed. Qualitative themes of written responses were also examined.

**Results:**

Subjects (*N* = 98) were 26–57 years old (mean 40 years). Three-quarters (76%) were female. The percentage always wearing a helmet riding bicycles was 63% (subjects), 58% (spouses/partners), and 51% (children), compared to 11, 14 and 37% on ATVs, respectively. Moreover, the percentage never wearing a helmet while on an ATV was 68% for subjects, 71% for spouses, and 47% for children. Despite helmet use differences between bicycles and ATVs, the importance of children wearing a helmet on these vehicles was rated highly and equally important, 9.28 and 9.58 on a 1–10 scale, respectively. Higher proportions of subjects’ oldest children wore a bike helmet 100% of the time if at least one parent always wore a helmet (81%), compared to children whose parents both wore helmets < 100% of the time or didn’t ride (21%) (*p* < 0.0001). The proportion of children wearing ATV and bicycle helmets less than 100% of the time was significantly higher if parents reported barriers to effectively enforcing helmet use than if they did not (*p* = 0.04 and *p* = 0.004, respectively). Many reported a “strict no helmet, no bike/ATV riding rule” as being most effective in getting their children to always wear a helmet.

**Conclusions:**

This study is the first to explore family helmet use while riding bicycles vs ATVs. Although parent’s belief in the importance of helmet use was high for both, helmet use was greater when riding bicycles. Further research is needed to better understand the social and environmental influences that shape parental helmet attitudes and practices in order to improve safety interventions for increasing pediatric helmet use.

## Background

Bicycle and all-terrain vehicle (ATV) riding are common childhood recreational activities, even though the American Academy of Pediatrics does not recommend children less than 16 years old ride ATVs (American Academy of Pediatrics Committee on Injury and Poison Prevention, 2000). Although studies by the Centers for Disease Control and Prevention (CDC) indicate less bicycle riding has played a considerable role in the decrease in bicycle-related injuries and deaths among youth over the past few decades, helmet use may also be a significant contributor (Vargo et al., 2015). Despite this decrease, a study in 2010 reported children 5–14 years old still had the highest rate of bicycle-related injuries among all ages in the United States (U.S.) (Dellinger & Kresnow, 2010).

Although bicycle use is more prevalent than ATV use, more children < 16 years old die in the U.S. each year from ATV-related events than from bicycle crashes (Helmkamp et al., 2009). Children < 16 years old account for about one-fifth of all U.S. ATV-related fatalities, and it is estimated about four children in this age range are seen in an emergency department with an ATV-related injury every hour (U.S. Consumer Product Safety Commission, 2019). Moreover, children and adolescents have ~ 4.5 times greater risk of ATV-related injury compared to middle-aged adults (U.S. Consumer Product Safety Commission., 1998).

Both bicycle and ATV crashes can result in severe traumatic brain injury (Dellinger & Kresnow, 2010; National Center for Injury Control, Centers for Disease Control and Prevention, 1995; Denning et al., 2013a; Denning et al., 2014; Denning & Jennissen, 2018; Denning et al., 2013b), yet helmet use among children riding bicycles (Ehrlich et al., 2001; Finnoff et al., 2001; Hoye, 2018) and on ATVs (Denning et al., 2014; Denning & Jennissen, 2018) remains low. The specific factors contributing to children’s helmet use are poorly understood. An earlier survey study related to bicycle helmets suggested parental role modeling and supervision may be important contributors to children wearing helmets (Ehrlich et al., 2001). Our study’s objective was to directly compare family helmet use for ATVs versus bicycles, something not previously reported. In addition, we determined the attitudes towards, and the level of importance ascribed by parents/guardians for the use of ATV and bicycle helmets.

## Methods

### Study population

A survey was administered to a convenience sample of adult attendees at a university-sponsored health fair in 2017 by the Iowa ATV Injury Prevention Task Force (https://uichildrens.org/health-library/all-terrain-vehicle-atv-safety). Attendees at the health fair were primarily employees of the university, particularly the hospital. Inclusion criteria were that the participant be a parent/guardian with at least one child less than 18 years old still living at home, and that one or more of these children rode a bicycle and/or an ATV in the past year. Ninety-eight attendees meeting these criteria completed the survey. For ease of reading, parents/guardians will subsequently be referred to in this report as “parents.” As the study’s analysis was performed on an existing dataset that had been collected anonymously, the authors’ Institutional Review Board deemed this study exempt.

### Survey

An ATV was defined as a vehicle with a straddle seat, handlebars for steering and low-pressure tires, and a picture of an ATV was provided on the survey (see the Additional File for the survey in its entirety). The survey was developed, including attitudinal questions and their response options, through a collaborative and iterative process among members of the Iowa ATV Injury Prevention Task Force and bicycle safety experts at the University of Iowa Stead Family Children’s Hospital. Demographic variables for those surveyed included age, sex, where they lived (farm, in the country but not on a farm, in town), and how many children under 18 years old currently lived in their home. Information collected about family members included the age and sex of the participant’s spouse/partner and of minors living at home.

Questions related to ATVs with categorical answers included whether the family currently owned an ATV, whether persons in the home had ridden an ATV in the past year, and if so, what was their frequency of helmet use: 0% (Never), 1–25%, 26–50%, 51–75%, 76–99% or 100% (Always) of the time. If any persons in the home had ridden an ATV in the past year, respondents were asked, “On a scale of 1-10, how important do you think it is for your child/children to wear a helmet when riding on an ATV?” Answers were on a Likert type scale with 1 designated as “Not at all important” and 10 as “Very important.” They were also asked two attitude-related questions. A series of questions similar to the ATV-related questions were then asked about family bicycle helmet use.

For the attitude-related questions regarding their children’s helmet use, subjects were provided multiple response options to which they could select all that apply including “Other.” The first attitude query stated, “I don’t feel they always need to wear a bicycle/ATV helmet because:” and a response example being, “They only ride in places I think are safe.” The other stated, “I would like them to always wear a bicycle/ATV helmet, but factors that decrease my effectiveness in enforcing helmet use include:” and a response example being “I do not have appropriate helmets for them to use.” An additional open-ended question was, “Have you found an effective way to get your child/children to always wear a helmet when they ride on a bicycle/ATV?”

### Quantitative analysis

The survey was administered on paper, and data were entered into Qualtrics™. Aggregate results were then exported as an Excel spreadsheet and imported into IBM SPSS Statistics, previously named SPSS (Statistical Package for the Social Sciences), for descriptive (frequencies) and bivariate (Fisher’s exact test) analyses. All *p*-values were two-tailed. The helmet use frequency of the respondent’s oldest child < 18 years old who lived at home and had ridden a bicycle and/or ATV was used in comparative analyses. Missing data were not included in analyses.

### Qualitative analysis

Responses to open-ended questions were compiled. Themes and sub-themes were independently identified by three research team members. The research team then reviewed and discussed the coding. An iterative process was used to resolve all differences. The total number of comments was recorded and the number of comments for each theme and sub-theme were determined. Representative comments are included in this report.

## Results

### Demographics and helmet use

There were 98 survey participants included in analysis, with 83 spouses/partners, and 175 minor children living at home (Table 1). The highest proportion of respondents were 36–45 years old and nearly four-fifths (78%) were female. There were roughly equal proportions of children in the three age groups (0–5, 6–11, and 12–17 years old) and the same proportions by sex. Answers to “where do you live” were: 5% on a farm, 9% in the country but not on a farm, and 86% in town. Nineteen percent of families (18/98) owned an ATV.

**Table 1:** Demographic characteristics and helmet use of study participants^a^ and their families

	Respondents n (column %) ^b^	Spouse/partner n (column %) ^b^	Minor children [Age range] n (column %) ^b^
Group N	98	83	175
Age			
26–35 years	22 (22)	18 (23)	[0–5 years] 53 (32)
36–45 years	59 (61)	52 (52)	[6–11 years] 59 (35)
46–60 years	17 (17)	20 (25)	[12–17 years] 56 (33)
Sex			
Male	22 (22)	61 (78)	77 (50%)
Female	76 (78)	17 (22)	78 (50%)
Rode an ATV in past year			
Yes	19 (19)	14 (17)	43 (25)
No	79 (81)	69 (83)	132 (75)
ATV helmet use past year			
0% (Never)	13 (68)	10 (71)	20 (47)
1–25%	1 (5)	1 (7)	2 (5)
26–50%	2 (11)	0 (0)	3 (7)
51–75%	1 (5)	1 (7)	0 (0)
76–99%	0 (0)	0 (0)	2 (5)
100% (Always)	2 (11)	2 (14)	16 (37)
Rode a bicycle in past year			
Yes	65 (66)	50 (60)	135 (77)
No	33 (34)	33 (40)	40 (23)
Bicycle helmet use past year			
0% (Never)	13 (22)	11 (22)	11 (8)
1–25%	1 (2)	2 (4)	15 (1)
26–50%	2 (3)	2 (4)	6 (4)
51–75%	2 (3)	3 (6)	8 (6)
76–99%	4 (6)	3 (6)	26 (19)
100% (Always)	37 (63)	29 (58)	69 (51)

^a^Subject inclusion criterion were adults that had one or more children < 18 years old living at home and that one or more of the children had ridden a bicycle and/or on an all-terrain vehicle (ATV) in the past year

^b^Total of column n values by category may not equal total Group N because of missing data

For ATV helmet use, the proportion of adults who never wore helmets (70%) was higher than their minor children (47%). Only 12% of all adults (respondents and spouse/partners) always wore ATV helmets compared to 37% of child riders. Overall helmet use riding bicycles was significantly higher than for ATVs; over half (135/244, 55%) of family members always wore helmets when riding bicycles versus a quarter (20/76, 26%) when on ATVs (*p* < 0.01).

### Comparisons of helmet use

When ATV and bicycle helmet use was compared for each riding population, statistically significant differences were observed (Table 2). We found the proportion of respondents, spouses/partners, and children who always wore bicycle helmets was approximately 6, 4, and 1.4 times higher, respectively, than comparable values for ATV helmet use. Conversely, the proportion that never wore a helmet was markedly higher among ATV riders than among bicyclists. A higher percentage of children wore a helmet more than three-quarters of the time on bicycles (95/135, 70%) as compared to ATVs (18/43, 42%) (*p* = 0.001).

**Table 2:** Comparison of helmet use frequencies for ATVs and bicycles among participants and their family members

	Respondents			Spouses/Partners			Children		
	ATV n (col%)	Bicycle n (col%)	*p*-value^a^	ATV n (col%)	Bicycle n (col%)	*p*-value^a^	ATV n (col%)	Bicycle n (col%)	*p*-value^a^
Group N	19	59		14	50		43	135	
Helmet use									
0% Never	13 (68)	13 (22)	< 0.0001	10 (71)	11 (22)	0.01	20 (47)	11 (8)	< 0.0001
1–99%	4 (21)	9 (15)		2 (14)	10 (20)		7 (16)	55 (41)	
100% Always	2 (11)	37 (63)		2 (14)	29 (58)		16 (37)	69 (51)	

^a^Fisher’s exact test

Parental helmet use was also directly compared to their oldest child’s for ATVs and/or bicycles. In homes where parents rode ATVs, but never wore helmets, nearly three-quarters (8/11) of their oldest children also never wore a helmet. If both parents always wore their helmet on ATVs, then the child did as well (2/2, 100%). In a similar comparison for bicycles, 44% (4/9) of oldest children whose parents never wore a bicycle helmet, also rode un-helmeted all the time. Moreover, higher proportions of the oldest child wore a bicycle helmet 100% of the time if at least one parent always wore a helmet (29/36, 81%), compared to children whose parents both wore helmets less than 100% of the time or didn’t ride (8/38, 21%) (*p* < 0.0001).

### Importance of wearing helmets

Respondents were asked to rank the importance of wearing helmets on a scale of 1–10, where 1 was “Not at all important” and 10 was “Very important” (data not shown in tables). The mean (SD) for ATV and bicycle helmets was 9.58 (1.21) and 9.28 (1.60), respectively. No one ranked the importance of wearing a helmet as < 4 for either vehicle. Among respondents who had at least one child who had ridden on an ATV in the past year (*n* = 24), 88% said helmet use was “very important” (rank = 10), 8% ranked it 7–9, and 4% ranked it 4–6. Among subjects who had at least one child who had ridden bicycles in the past year (*n* = 74), 77% said helmet importance was a 10, 14% ranked it 7–9, and 9% selected 4–6. These values were not significantly different from the values for ATVs (*p* = 0.69).

### Attitudinal barriers to children’s helmet use

Among participants who selected a response to the question “I don’t feel they always need to wear a helmet because:” half of them selected “They don’t ride very often” for ATVs, while only 10% indicated this for bicycles (*p* < 0.0001) (Table 3). Other responses with higher percentages for ATVs compared to bicycles regarding why they didn’t feel their child needed to wear a helmet included that they ride as a passenger with an adult who always rides safely (33% vs. 3%, *p* < 0.001), and that the subject or another responsible person watches them riding to make sure they are riding safely (21% vs. 10%, *p* < 0.01). Overall, fewer subjects provided responses to the statement, “I would like them to always wear a helmet, but factors that decrease my effectiveness in enforcing helmet use include.” The most common responses selected from those provided for ATVs and bicycles was, “I am not around when they ride” and “I do not have appropriate helmets for them to use.”

**Table 3:** Responses to the indicated statements by study participants

	ATVs n (%) ^a^	Bicycles n (%) ^a^
Group N	24	74
(a) I don’t feel they always need to wear helmet because:		
They don’t ride very often.	12 (50)	7 (10)
They only ride in places I think are safe.	6 (25)	13 (18)
They ride as a passenger with an adult who always rides safely.	8 (33)	2 (3)
I or another responsible person watch them riding to make sure they are riding safely.	9 (21)	7 (10)
They have been riding for some time without a serious injury and I trust they can ride safely.	4 (17)	6 (8)
I think crashes happen because people ride recklessly, and I make sure my children do not.	3 (13)	6 (8)
Other	1 (4)	3 (4)
(b) I would like them to always wear a helmet, but factors that decrease my effectiveness in enforcing helmet use include:		
My spouse/significant other is not supportive of enforcing helmet use.	0 (0)	1 (1.3)
I do not have appropriate helmets for them to use.	4 (17)	4 (5)
The children they ride with don’t wear helmets.	0 (0)	2 (3)
I am not around when they ride.	4 (17)	9 (12)
My children will not listen to me when I tell them to wear a helmet.	1 (4)	1 (1.3)
I do not have specific consequences for when my children don’t wear a helmet.	0 (0)	2 (3)
I have problems enforcing the consequences for when my children don’t wear a helmet.	1 (4)	2 (3)
There are no helmet laws requiring them to wear helmets.	0 (0)	1 (1.3)
Other	6 (25)	1 (1.3)

^a^Total of column n values by category may not equal total Group N because participants could enter more than one response to each statement or did not provide a response if they did not agree with the statement. The percentage noted in parentheses is that of Group N

Comparisons of helmet use by the oldest resident child were made based on whether participants selected responses to the attitudinal survey statements (Table 4). Subjects who selected responses to the statement as to why they felt their child didn’t always need to wear a bicycle helmet had higher percentages of children who did not wear a helmet 100% of the time, compared to parents who did not select responses (*p* < 0.0001). Similarly, participants who identified barriers to effectively enforcing both ATV and bicycle helmet use had higher percentages of children who did not always wear helmets versus parents that did not identify barriers (*p* = 0.04 and *p* = 0.004, respectively).

**Table 4:** Comparison of helmet use by the oldest child < 18 years old of participants who did or did not select responses to the indicated survey statements

		Helmet use (% of time)		
		< 100% n (col%)	100% n (col%)	*p*-value^a^
ATV group *N* = 24				
Indicated child doesn’t always need to wear a	Yes	12 (80)	4 (44)	0.18
helmet^b^	No	3 (20)	5 (56)	
Identified barriers to effective enforcement of	Yes	12 (80)	3 (33)	0.04
helmet use^c^	No	3 (20)	6 (67)	
Bicycle group *N* = 74				
Indicated child doesn’t always need to wear a	Yes	23 (58)	2 (6)	< 0.0001
helmet^b^	No	17 (43)	32 (94)	
Identified barriers to effective enforcement of	Yes	17 (43)	4 (12)	0.004
helmet use^c^	No	23 (58)	30 (88)	

^a^Fisher’s exact test

^b^I don’t feel they always need to wear a helmet because

^c^I would like them to always wear a helmet, but factors that decrease my effectiveness in enforcing helmet use include

### Qualitative results

Table 5 summarizes the themes and subthemes identified, as well as their frequency for responses to the question, “If you have found an effective way to get your child/children to always wear a helmet when they ride a bicycle/ATV, what is it?” There were only 12 responses provided for ATV helmet use, and nearly all were under the theme “Coercion,” specifically having a hard rule for helmet use. Statement examples included, “If they don’t wear one, they don’t get to ride,” “If they don’t have a helmet on, they don’t get the keys,” and “I just make them.”

**Table 5:** Qualitative analysis of participant’s responses regarding effective ways to get their child/children to wear a helmet

Question: If you have found an effective way to get your child/children to always wear a helmet when they ride a bicycle/ATV, what is it?		
Theme & subthemes	ATV n (col%)	Bicycle n (col%)
Theme: Coercion		
No helmet, no riding	7 (70)	18 (55)
Hard rule, non-negotiable	3 (30)	8 (24)
Negative consequences for violation	0	7 (21)
Theme total	10	33
Theme: Social encouragement		
Role modeling		6 (60)
Frequent reminders		3 (30)
Positive reinforcement		1 (10)
Theme total	0	10
Theme: Habit formation		
Start early		4 (67)
Specific strategy to encourage habit		2 (33)
Theme total	0	6
Miscellaneous	2	2

Relative to ATVs, more strategies were identified for effective means of getting their child/children to always wear a helmet when bicycling, with a total of 51 responses. These responses were under the themes of “Coercion” (65%), “Social encouragement” (20%), and “Habit formation” (12%). In terms of coercion, many of the survey participants specifically stated the need for a strict “no helmet, no riding rule,” and that helmet use was mandatory if their children wanted to ride a bicycle. One stated, “It has been non-negotiable since day one and is now not even a second thought.” Others stated the use of negative consequences such as losing the privilege to ride and threatening to “take away electronics.”

For social encouragement, six of the ten responses involved the importance of role modeling, and others mentioned frequent reminders and reinforcing the importance of wearing a helmet. Another incentive strategy was “buying them the helmet they wanted.” Regarding habit formation, respondents stated it was essential to start early, “put a helmet on them from the very beginning” and “start at a young age with riding toys.” One parent developed a helmet wearing habit by having their child always hang the helmet on the bike.

## Discussion

### ATV/bicycle helmet use and importance

Our study is the first to directly compare bicycle and ATV helmet use in families, and to explore the level of importance parents ascribe to wearing helmets on these vehicles. We found significantly greater bicycle helmet use in families as compared to ATVs, although parental belief in the importance of helmet use was equally high for both. This is an interesting finding not previously reported in the literature.

Previous studies have shown that parents who are aware of an important child safety practice do not always implement them, such as properly protecting children in car seats (Yanchar et al., 2012; Yanchar et al., 2015). Additionally, a national SAFE KIDS study found parents recognized the serious potential danger of children participating in water activities (Cody et al., 2004), and nearly all (94%) stated they supervised their children while they were swimming. However, the organization was alarmed that parents reported concurrently participating in a number of distracting behaviors during supervision including talking to others (38%), reading (18%), eating (7%), talking on the phone (11%), and closing eyes and relaxing (4%). The disconnect between parental attitudes that helmet use is very important and their lack of ensuring their children wear helmets deserves further investigation.

### Parental Role Modeling

We found a positive association between children always wearing a helmet and parents who wore them as well. Other studies have also shown this positive relationship between parent and child helmet use (Ehrlich et al., 2001; Berg & Westerling, 2001). A 2012 study found children were more likely to wear a helmet while biking (90% vs. 38%), when parents reported always wearing a helmet (Jewett et al., 2016). Another study showed children riding bikes along with helmeted adults were over 2.5 times more likely to wear a helmet than those riding alone, and a higher proportion of children wore helmets if they were riding beside helmeted adults (95%) than with un-helmeted ones (41%) (Khambalia et al., 2005).

Moreover, direct comparisons showed the safety behaviors of children are more likely to be predicted by the safety behaviors of their parents, than by parental delivery of safety information (Morrongiello et al., 2008). A rural focus group study noted some parents could become motivated to role model helmet use if they thought it was valuable to have consistency between their personal behavior and expectations for their child (Robertson et al., 2014). Additionally, ATV riding adolescents felt they would be more likely to wear helmets if their parents set an example and used helmets (Adams et al., 2013). Thus, parents and other adults who do not wear helmets have a significantly negative impact on helmet use by children. Injury prevention efforts with direct messaging to children about the importance of helmet use should be paired with programs designed to increase helmet use by adults.

### Attitudinal barriers

Our study showed lower proportions of children always wore their helmet if their parents indicated there were reasons the children didn’t need to wear a helmet or if they identified barriers to effectively enforcing helmet use, compared to children whose parents did not have these attitudes. Some survey respondents indicated they didn’t feel their child always needed to wear a helmet, because they had been riding some time without a serious injury and that they could be trusted to ride safely. A focus group study of Arkansas parents and adolescents riding ATVs found many participants failed to recognize the possibility of a serious injury, long-term disability, or even death from an ATV crash (Adams et al., 2013). Many felt user experience reduced the likelihood of a crash. As a result, some parents only enforced rules such as wearing a helmet when children were inexperienced and no longer did so when they became adolescents (Robertson et al., 2014). However, we know that adolescents, especially males, will take more risks compared to younger children, and that higher proportions of adolescents have ATV-related crashes and injuries than those who are younger (Denning et al., 2014). Similar studies have shown many children, adolescents, and adults perceive riding a bicycle as not being very dangerous, and their risk of sustaining a head injury when bicycling without a helmet as “slight” (Finnoff et al., 2001; Ong et al., 2018).

Focus group studies also found many adults and teens were more worried about terrain issues, which could lead to a crash, than with the vehicles themselves (Adams et al., 2013). This thinking is illustrated by the one-fourth and one-third of respondents with ATV riding children in our study who stated they didn’t think their children always needed to wear a helmet because they only ride in areas that are “safe” and because they or another responsible person makes sure they are “riding safely,” respectively. Similarly, study respondents with bicycle-riding children affirmed the most frequent reason they didn’t think their child always needed to wear a bicycle helmet was that their child only rode in “safe” places. This has previously been found to be a significantly more common reason given by parents with younger children for not wearing a helmet compared to older youth (Miller et al., 1996).

### Helmet use facilitators

Respondents in our study stated the most effective way of getting their children to always wear a bicycle or ATV helmet was implementation of a strict, non-negotiable “no helmet, no riding rule”. A previous study found children whose families enforced firm rules regarding helmet use on bicycles had higher proportions who wore a helmet most or all of time (88%), as compared to children with a partial rule or no rule at all (19%) (Miller et al., 1996). In fact, the presence of a strict rule increased the likelihood of bicycle helmet use 46-fold. A focus group of adolescents indicated the most common reason they wore ATV helmets was that their parents or riding club mandated it (Adams et al., 2013). Other studies have shown parent-established rules mandating helmet use are a critical factor in a child’s decision to wear a helmet (Berg & Westerling, 2001; Khambalia et al., 2005; Keezer et al., 2007). Although teens may be less likely to adhere to a strict rule, adolescents whose parents make it a requirement to use a helmet still have higher proportions who wear a helmet, compared to those without parental rules (Berg & Westerling, 2001). In addition, helmet-wearing parents have stronger bicycle helmet rules for their experienced riding children than parents who do not wear helmets (Ross et al., 2014).

Those with child bicyclists in the study offer additional suggestions for ensuring child helmet use. These included enforcing negative consequences, starting helmet use early to establish it as a habit, and providing social encouragement including role modeling. Other studies have echoed some of these suggestions (Khambalia et al., 2005; Robertson et al., 2014), and have shown they can have positive effects on helmet use by children (O'Callaghan & Nausbaum, 2006). Focus groups felt ATV helmet use needed to “start at home,” and parents needed to enforce usage (Adams et al., 2013). Factors associated with bicycle helmet use by children include parental rules and involvement, parental helmet ownership and use, peer helmet use, and positive attitudes about helmets (Finnoff et al., 2001; Berg & Westerling, 2001; Miller et al., 1996; Cryer et al., 1998; Hu et al., 1994).

### Limitations

Our study involved a convenience sample of attendees at a university hospital sponsored health fair and had a relatively small sample size. In addition, demographic data, including race, ethnicity, and socioeconomic status were not obtained. This limits the generalizability of our results. There is also the possibility of recall bias in accurately stating the frequency of ATV and bicycle helmet use. In addition, survey studies have the possibility of responder or social desirability bias. However, the survey was completed anonymously which may help reduce this bias. Since it was reasoned that a shorter survey would increase participation in the context of the health fair, the survey was not able to further explore the relationship between the perceived importance of helmet use and the relative lack thereof. Nonetheless, to our knowledge, direct comparisons of family helmet use on ATVs versus bicycles have not previously been reported, and the specific factors contributing to helmet use in children is not completely understood. Our study helps fill some of that knowledge gap.

## Conclusions

This study is the first to directly compare family helmet use on ATVs and bicycles, as well as parental attitudes regarding the relative importance of wearing helmets on these vehicles. We found significantly higher helmet use with bicycles compared to ATVs, although parental beliefs in the importance of helmet use was similarly high for both. There was a positive association between children always wearing a helmet and parents who wore them as well, and a negative association between children’s helmet use and parents with negative attitudes towards helmet use and/or their ability to enforce it. However, some strategies to increase their child’s helmet use were identified, including setting strict rules, role modeling, and early helmet use. Additional research will be needed to fully understand the social and environmental influences that shape parental attitudes and practices in order to increase pediatric helmet use on both bicycles and ATVs.

## Supplementary information

**Additional file 1.** UI Health Fair Survey 2017. This file is the survey used at the University of Iowa Health Fair in 2017 to collect the data for this study.

## Data Availability

Data and materials are available to other parties for research purposes after a data sharing agreement plan is agreed to and signed.

## Abbreviations

ATV: All-terrain vehicle
CDC: Centers for Disease Control and Prevention
SPSS: Statistics Package for the Social Sciences
U.S.: United States
