# Supplementary material for: Parental attitudes and family helmet use for all-terrain vehicles and bicycles
Source: Inj Epidemiol. 2020 Jun 12;7(Suppl 1):23. doi: 10.1186/s40621-020-00253-2 (PMC7291627; doi:10.1186/s40621-020-00253-2)
Supplement: Supplementary file 1 — Additional file 1. UI Health Fair Survey 2017. This file is the survey used at the University of Iowa Health Fair in 2017 to collect the data for this study. [file 40621_2020_253_MOESM1_ESM.docx]

**UI HEALTH FAIR SURVEY 2017**

**Q1.** How old are you? I am ______________ years old

**Q2.** What gender are you? 🞎 Male 🞎 Female

**Q3.** Where do you live? 🞎 On a farm 🞎 In the country, but not on a farm 🞎 In town

**Q4.** How many children under the age of 18 currently live in your home? ___________ children

**
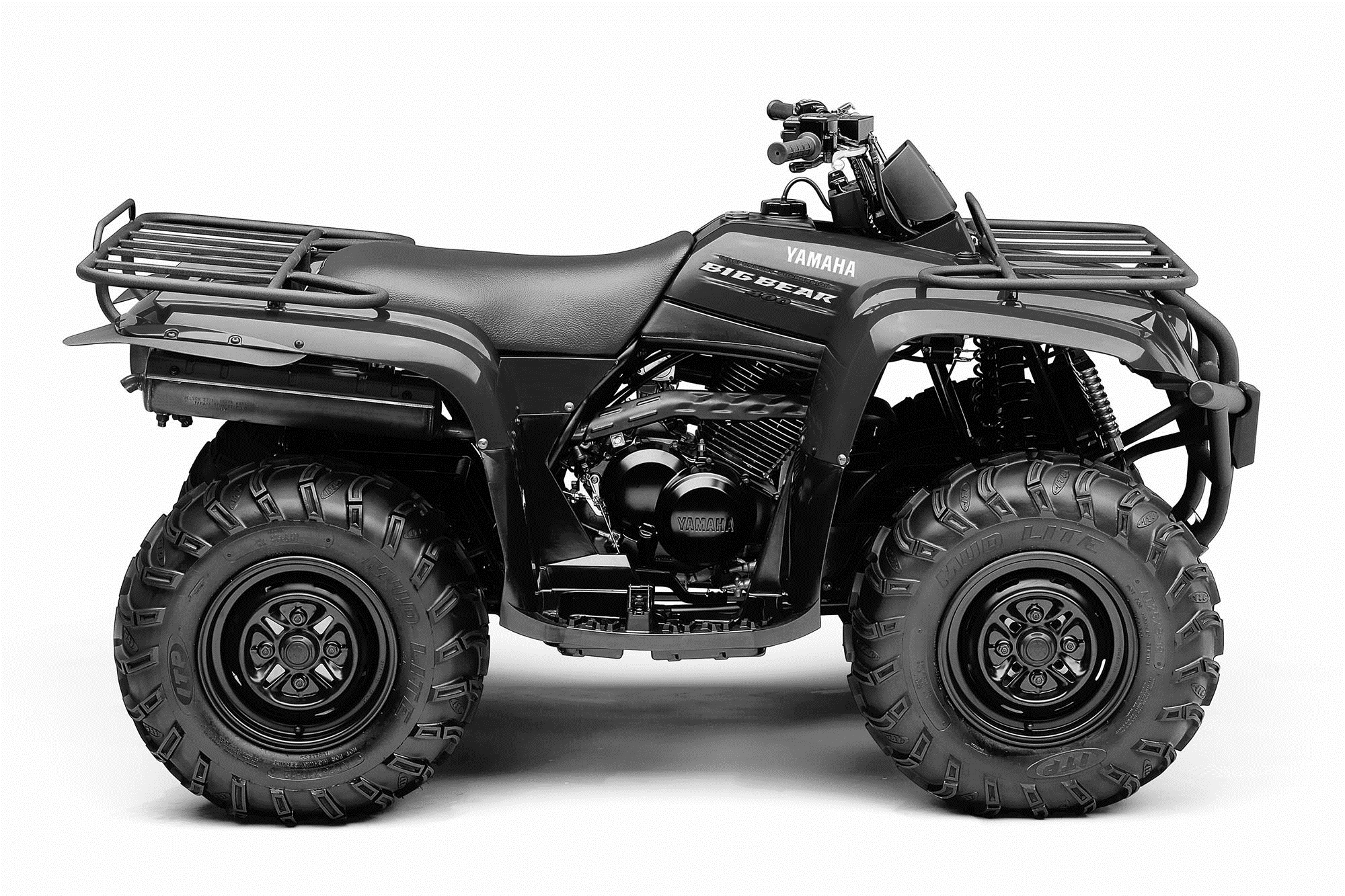
Fill out the rest of the survey for up to your 5 oldest children under the age of 18.**

**ALL-TERRAIN VEHICLE (ATV)**

**Q5.** Does your family currently own an ATV? 🞎 No 🞎 Yes

**Q6.** Please indicate how often persons living in your home have worn a helmet when riding on an ATV in the past year.

|  |  |  | | **Check percent of the time a helmet was worn for any persons who rode an ATV in the past year.** | | | | | |
| --- | --- | --- | --- | --- | --- | --- | --- | --- | --- |
|  | **Age (years)** | **Gender**  **(M/F)** | **Didn’t ride on an ATV in past year** | **0%**  **Never** | **1-25%** | **26-50%** | **51-75%** | **76-99%** | **100%**  **Always** |
| You |  |  |  |  |  |  |  |  |  |
| Spouse/Partner |  |  |  |  |  |  |  |  |  |
| Child 1 |  |  |  |  |  |  |  |  |  |
| Child 2 |  |  |  |  |  |  |  |  |  |
| Child 3 |  |  |  |  |  |  |  |  |  |
| Child 4 |  |  |  |  |  |  |  |  |  |
| Child 5 |  |  |  |  |  |  |  |  |  |

**If no one in your family has ridden an ATV in the past year, go to Question 10. Otherwise continue.**

**Q7.** On a scale of 1-10, how important do you think it is for your child/children to wear a helmet when riding on an ATV? Circle the number for your answer.

1 2 3 4 5 6 7 8 9 10

Not at all Very

important important

**Q8.** What thoughts do you have about helmet use by your children when they are riding on an ATV? Check all that apply. (Continued on other side)

***I don’t feel they always need to wear an ATV helmet because:***

☐ They don’t ride very often.

☐ They only ride in places I think are safe.

☐ I or another responsible person ride on the ATV with them and always ride safely.

☐ I or another responsible person watch them riding to make sure they are riding safely.

☐ They have been riding for some time without a serious injury and I trust they can ride safely.

☐ I think crashes happen because people ride recklessly and I make sure my children do not.

☐ Other: __________________________________________________________________________________________________

***I would like them to always wear an ATV helmet, but factors that decrease my effectiveness in enforcing helmet use include:***

☐ My spouse/significant other is not supportive of enforcing helmet use.

☐ I do not have appropriate helmets for them to use.

☐ The children they ride with don’t wear helmets.

☐ I am not around when they ride ATVs.

☐ My children will not listen to me when I tell them to wear a helmet.

☐ I do not have specific consequences for when my children don’t wear a helmet.

☐ I have problems enforcing the consequences for when my children don’t wear a helmet.

☐ There are no helmet laws requiring them to wear ATV helmets.

☐ Other: __________________________________________________________________________________________________

**Q9.** Have you found an effective way to get your child/children to always wear a helmet when they ride on an ATV? If so, please share.

|  |
| --- |
|  |

**BICYCLE**

**Q10.** Please indicate how often persons living in your home have worn a helmet when riding a bicycle in the past year.

|  |  |  | | **Check percent of the time a helmet was worn for any persons who rode a bicycle in the past year.** | | | | | |
| --- | --- | --- | --- | --- | --- | --- | --- | --- | --- |
|  | **Age (years)** | **Gender**  **(M/F)** | **Didn’t ride a bicycle in past year** | **0%**  **Never** | **1-25%** | **26-50%** | **51-75%** | **76-99%** | **100%**  **Always** |
| You |  |  |  |  |  |  |  |  |  |
| Spouse/Partner |  |  |  |  |  |  |  |  |  |
| Child 1 |  |  |  |  |  |  |  |  |  |
| Child 2 |  |  |  |  |  |  |  |  |  |
| Child 3 |  |  |  |  |  |  |  |  |  |
| Child 4 |  |  |  |  |  |  |  |  |  |
| Child 5 |  |  |  |  |  |  |  |  |  |

**If no one in your family has ridden a bicycle in the past year, you have finished the survey. Thank You. Otherwise continue.**

**Q11.** On a scale of 1-10, how important do you think it is for your child/children to wear a helmet when riding a bicycle? Circle the number for your answer.

1 2 3 4 5 6 7 8 9 10

Not at all Very

important important

**Q12.** What thoughts do you have about helmet use by your children when they are riding a bicycle? Check all that apply.

***I don’t feel they always need to wear a bicycle helmet because:***

☐ They don’t ride very often.

☐ They only ride in places I think are safe.

☐ They ride as a passenger with an adult who always rides safely.

☐ I or another responsible person watch them riding to make sure they are riding safely.

☐ They have been riding for some time without a serious injury and I trust they can ride safely.

☐ I think crashes happen because people ride recklessly and I make sure my children do not.

☐ Other: __________________________________________________________________________________________________

***I would like them to always wear a bicycle helmet, but factors that decrease my effectiveness in enforcing helmet use include:***

☐ My spouse/significant other is not supportive of enforcing helmet use.

☐ I do not have appropriate helmets for them to use.

☐ The children they ride with don’t wear helmets.

☐ I am not around when they ride.

☐ My children will not listen to me when I tell them to wear a helmet.

☐ I do not have specific consequences for when my children don’t wear a helmet.

☐ I have problems enforcing the consequences for when my children don’t wear a helmet.

☐ There are no helmet laws requiring them to wear bicycle helmets.

☐ Other: __________________________________________________________________________________________________

**Q13.** Have you found an effective way to get your child/children to always wear a helmet when they ride a bicycle? If so, please share.

|  |
| --- |
|  |

**THANK YOU FOR COMPLETING OUR SURVEY**
